# Metabolic syndrome and associated factors in Iranian children and adolescents: the CASPIAN-V study

**DOI:** 10.15171/jcvtr.2018.37

**Published:** 2018-12-05

**Authors:** Ramin Heshmat, Zeinab Hemati, Mostafa Qorbani, Laleh Nabizadeh Asl, Mohammad Esmaeil Motlagh, Hasan Ziaodini, Majzoubeh Taheri, Zeinab Ahadi, Gita Shafiee, Tahereh Aminaei, Hooman Hatami, Roya Kelishadi

**Affiliations:** ^1^Chronic Diseases Research Center, Endocrinology and Metabolism Population Sciences Institute, Tehran University of Medical Sciences, Tehran, Iran; ^2^Pediatrics Department, Child Growth and Development Research Center, Research Institute for Primordial Prevention of Non-communicable Disease, Isfahan University of Medical Sciences, Isfahan, Iran; ^3^Non-Communicable Diseases Research Center, Alborz University of Medical Sciences, Karaj, Iran; ^4^Endocrinology and Metabolism Research Center, Endocrinology and Metabolism Clinical Sciences Institute, Tehran University of Medical Sciences, Tehran, Iran; ^5^Department of Nutrition and Dietetics, Faculty of Health Sciences, Hacettepe University, Ankara, Turkey; ^6^Pediatrics Department, Ahvaz Jundishapur University of Medical Sciences, Ahvaz, Iran; ^7^Bureau of Health and Fitness, Ministry of Education and Training, Tehran, Iran; ^8^Office of Adolescents and School Health, Ministry of Health and Medical Education, Tehran, Iran; ^9^Student Research Committee, Alborz University of Medical Sciences, Karaj, Iran

**Keywords:** Metabolic Syndrome, Obesity, Pediatric

## Abstract

***Introduction:***Metabolic syndrome (MetS) is one of the common metabolic disorders seen in children and adolescents. This study aims to assess the rate of the MetS and its associated factors in a nationally-representative sample of Iranian pediatric age groups.

***Methods:*** This nationwide cross- sectional study was designed in 2015 in 30 provinces of Iran. Participants consisted of 4,200 school students, aged 7-18 years, studied in a national school-based surveillance program (CASPIAN-V). Physical examination and laboratory tests were performed using standard protocols. Blood samples were drawn from 3834 students for biochemical tests.

***Results:*** The participation rate for blood sampling was 91.5%. MetS was significantly more prevalent among students in urban than in rural areas (5.7% vs. 4.8%, *P* value < 0.01). MetS was more prevalent in students with obese parents than in those with non-obese parents (6.4% vs. 4.5%, *P* value < 0.05). Significant association existed between moderate level of healthy nutritional behaviors and MetS after controlling for potential confounders (odds ratio [OR]: 0.62, 95% CI: 0.40-0.98). Students with high unhealthy nutritional behaviors showed an increased risk of MetS in crude (OR: 1.6, 95% CI: 1.05-2.44) and adjusted model (OR: 1.65, 95% CI: 1.05-2.63).

***Conclusion:*** High rate of MetS and associated risk factors was observed in Iranian pediatric age groups, with higher rates among boys. These findings provide useful information for effective preventive strategies based on diet, exercise, and lifestyle modification rather than therapeutic modalities.

## Introduction

The prevalence of non-communicable diseases (NCDs) is rapidly increasing worldwide, with special concern in developing communities. It estimated that NCDs will account 75% of global mortality rate especially in low-and middle- income countries.^1^ According to the Global Burden of Disease Study metabolic risk factors are the most important determinants of emerging problem of NCDs at global level.^2,3^

Metabolic syndrome (MetS) is one of the most common metabolic disorders, which leads to many NCDs as cardiovascular diseases, diabetes mellitus, some cancers, kidney disease, and mental disorders. The concept of MetS in pediatric group gained great concern during last decades because of various factors including gene-environment interactions, epidemiologic transition, nutritional disorders, sedentary lifestyle, and escalating trend of childhood obesity.^4,5^

Previous studies suggested that generalizes and abdominal obesity, weight disorder,^6^ dietary factors,^7^ sedentary lifestyle^8,9^ are the most of risk factors for MetS. According to various definitions of MetS, the rate of MetS in the pediatric age group, ranges between 0% to 19.2%.^10^

Although MetS has been extensively studied in adults, but limited evidence exists in pediatric group. Furthermore, considering that MetS tracks from childhood to adulthood, early detection of MetS prevent from subsequence clinical complications.^11^ In recent decades, Iran due to rapid epidemiological transition state is facing double burden of weight disorder, and MetS is documented even in young Iranian children.^12^

Present study aims to determine the prevalence of the MetS and its associated factors in a nationally representative sample of Iranian pediatric age groups.

## Materials and Methods

This national wide cross-sectional study was designed in 2015 as the fifth survey of a national surveillance program entitled “Childhood and Adolescence Surveillance and Prevention of Adult Non-communicable Disease (CASPIAN-V) study. This study was accomplished among aged 7–18 year-old students in urban and rural regions of 30 provinces of Iran, by via stratified cluster sampling method.^13^ There were 48 clusters in each province and 10 statistical units in each cluster, so a total of 14 400 students were participated in this study. In each province, randomly 14 clusters out of 48 clusters were selected for blood sample test and overall 4200 students were selected foe biochemical test. Data for some variables were missing.

Sample size was estimated according to previous published study in Iranian children and adolescentn,^14^ and using one proportion estimate formula for sample size and considering this formula the prevalence of MetS was considered as 4% , α- error 5%, and precision was considered 0.6%, that sample size was 14 400 students.

Study objectives and protocol were clarified prior to obtaining the informed written and verbal consents from the parents and students. A comprehensive description of the protocol has been described previously.^13^

### Procedure and measurements

Data were collected by the translated and validated questionnaire of the World Health Organization-Global School Students Health Survey (WHO-GSHS).^15^

Physical examination including weight and height was done under standard anthropometric. Weight was measured to the nearest 0.1 kg using a calibrated scale placed on a flat ground and height was measured to the nearest 0.1 cm using a port­able audiometer.^16^ Body mass index (BMI) was calculated as weight (kg) divided by height squared (m^2^). Waist circumference was measured to the nearest 0.1 cm three times and the average of three values was used for the analyses. A non-elastic tape was used to measure waist circumference at a point midway between the lower border of the rib cage and the iliac crest at the end of normal expiration.^17^ A mercury sphygmomanometer was used to measure BP on the right arm while participants were in the sitting position. Blood pressure (BP) was measured 2 times at 5 min intervals, and the average of the two values was used for the analyses.^18^

After 12 hours overnight fasting, venous blood sample was collected from students. Fasting blood glucose, triglycerides (TGs), total cholesterol (TC), low-density lipoprotein cholesterol (LDL-C) and high-density lipoprotein cholesterol (HDL-C) were measured enzymatically by Hitachi Auto Analyzer (Tokyo, Japan).^19,20^

### Definition of terms

The screen time (ST) behaviors was asked the numbers of hours that students spent watching television (TV) and/or videos, personal computer, or electronic games per day. Low ST was defined as less than 2 hours per day, and high ST was defined as a spending more than 2 hours per day for watching TV, and/or videos, personal computer, or electronic games.^21^

SES was calculated according previous study.^22^ SES was constructed using principle component analysis (PCA) method and considering some variables including father’s job and education, mother’s job and education, having private car and computer, and type of student’s school (private, public). SES was categorized as a tertile and the lowest tertile considered as a low SES and the third tertile as a high SES.

To define healthy and unhealthy nutritional behaviors, students were asked to determine frequency of consumption of breakfast, fruit, vegetables, milk, sugar sweetened beverages, fast foods, sweets and salty snacks. According to PCA method,^23^ two factors were loaded in PCA method; in the first factor which defined as healthy eating behavior consumption and in the second factor which defined as unhealthy eating behavior. Healthy and unhealthy eating behaviors factor was categorized into tertiles. The first tertile was defined as a low, second tertile as a moderate and third tertile as a high.

Validated questionnaire was used to measure physical activity (PA).^24^ Physical activity was categorized into two groups. The first group was defined as a low, second group as a high.

MetS was defined according to modified definition ATP III for children. Students were categorized as having MetS if they had more than three following criteria: fasting TG ≥150 mg/dL; HDL cholesterol ≤40 mg/dL; waist circumference (WC) ≥90th percentile for age and sex, according to national reference curves; SBP and/or DBP >90th percentile for sex, age and height, from national reference cut-off points; and FBG ≥100 mg/dL. LDL-C ≥ 110 mg/dL, TC ≥200 mg/dL, overweight and obesity also were determined as risk factors of MetS.^25^ Obesity was defined according WHO chart and students were categorized overweight and obese if MBI > 85th–95th, and BMI >95th, respectively.^26^

### Statistical analysis

Categorical variables were reported as frequency (percent). Chi-square test was used to assess association between categorical variables. Association between independent variables with MetS was assessed using two different logistic regression models: In model I crud association and in model II was adjusted for age, sex, region, SES, ST, PA, and BMI. All statistical analysis was performed using STATA version 11.0 and significant level was considered as *P* value < 0.05.

## Results

The participation rate of students for blood sample was 91.5% (3844 out of 4200 students selected for blood sampling). Totally 49.4% of students were girls and 71.4% lived in urban areas. The mean (SD) age of students was 12.3 ±3.2 years. The frequency of demographic and nutritional characteristics of participants was shown in Table 1.

**Table 1:** Frequency of demographic and nutritional characteristics: the CASPIAN -V study

Variable		No.	%
Sex	Male	2013	52.4
	Female	1831	47.6
	Missing	0	0
Age (y)	7-10	1147	29.8
	11-14	1655	43.1
	15-18	1042	27.1
	Missing	0	0
Region	Urban	2776	72.2
	Rural	1068	27.8
	Missing	0	0
PA	Low	2229	58.0
	High	1584	41.2
	Missing	32	0.8
SES	Low	1244	32.4
	Moderate	1220	31.7
	High	1205	31.3
	Missing	175	4.6
ST	Low	3226	83.9
	High	518	13.5
	Missing	100	2.6
Healthy nutritional behaviors	Low	1046	27.2
	Moderate	1016	26.4
	High	1057	27.5
	Missing	725	18.9
Unhealthy nutritional behaviors	Low	988	25.7
	Moderate	1106	28.8
	High	1025	26.7
	Missing	725	18.9
Parental obesity	Yes	1365	35.5
	No	2419	62.9
	Missing	60	1.6
MetS	Yes	188	4.9
	No	3544	92.2
	Missing	112	2.9

PA: physical activity; ST: screen time; SES: socioeconomic status; MetS, Metabolic syndrome.

Table 2 showed prevalence of MetS according to demographic and nutritional characteristics. The prevalence of MetS was significant higher in urban areas than rural areas (5.7% vs. 4.8%, 𝑃 < 0.01). Students with obese parents had higher frequency of MetS than those with non-obese parents (6.4% vs. 4.5%, 𝑃 < 0.05). The greater number of MetS components was significantly associated with gender, age, region and healthy nutritional behaviors in students (𝑃 < 0.05). Boys compared to girls and students in urban areas compared to rural areas had higher number of MetS components (𝑃 < 0.05). ST, SES, Unhealthy nutritional behaviors, parental obesity and PA were not associated with the number of MetS components (𝑃 > 0.05).

**Table 2:** Prevalence of metabolic syndrome according to demographic and nutritional characteristics: the CASPIAN -V study

Variable		MetS		Number of MetS components			
		Yes	No	0	1	2	3<
Sex	Male	108 (5.5)	1856 (94.5)	706 (35.9)	747 (38.0)	403 (20.5)	108 (5.5)
	Female	80 (4.5)	1688 (95.5)	737 (41.7)	610 (34.5)	341 (19.3)	80 (4.5)
	*P* value*	0.174		0.004			
Age (y)	7-10	55 (4.9)	1061 (95.1)	487 (43.6)	391 (35.0)	183 (16.4)	55 (4.9)
	11-14	85 (5.3)	1524 (94.7)	628 (39.0)	580 (36.0)	316 (19.6)	85 (5.3)
	15-18	48 (4.8)	959 (95.2)	328 (32.6)	386 (38.3)	245 (24.3)	48 (4.8)
	*P* value*	0.825		<0.001			
Region	Urban	154 (5.7)	2560 (94.3)	1008 (37.1)	992 (36.6)	560 (20.6)	154 (5.7)
	Rural	34 (3.3)	984 (96.7)	435 (42.7)	365 (35.9)	184 (18.1)	34 (3.3)
	*P* value*	0.004		0.001			
PA	Low	122 (5.6)	2046 (94.4)	822 (37.9)	788 (36.3)	436 (20.1)	122 (5.6)
	High	66 (4.3)	1474 (95.7)	612 (39.7)	558 (36.2)	304 (19.7)	66 (4.3)
	*P* value*	0.067		0.257			
SES	Low	66 (5.5)	1143 (94.5)	480 (39.7)	415 (34.3)	248 (20.5)	66 (5.5)
	Moderate	59 (5.0)	1122 (95.0)	451 (38.2)	453 (38.4)	218 (18.5)	59 (5.0)
	High	55 (4.7)	1121 (95.3)	450 (38.3)	427 (36.3)	244 (20.7)	55 (4.7)
	*P* value*	0.680		0.453			
ST	Low	159 (5.1)	2980 (94.9)	1220 (38.9)	1136 (36.2)	624 (19.9)	159 (5.1)
	High	26 (5.2)	475 (94.8)	191 (38.1)	182 (36.3)	102 (20.4)	26 (5.2)
	*P* value*	0.906		0.988			
Healthy nutritional behaviors	Low	61 (5.9)	974 (94.1)	391 (37.8)	349 (33.7)	234 (22.6)	61 (5.9)
	Moderate	42 (4.2)	961 (95.8)	421 (42.0)	352 (35.1)	188 (18.7)	42 (4.2)
	High	51 (4.9)	987 (95.1)	395 (38.1)	395 (38.1)	197 (19.0)	51 (4.9)
	*P* value*	0.207		0.036			
Unhealthy nutritional behaviors	Low	36 (3.7)	932 (96.3)	365 (37.7)	360 (37.2)	207 (21.4)	36 (3.7)
	Moderate	59 (5.4)	1036 (94.6)	432 (39.5)	399 (36.4)	205 (18.7)	59 (5.4)
	High	59 (5.8)	954 (94.2)	410 (40.5)	337 (33.3)	207 (20.4)	59 (5.8)
	*P* value*	0.077		0.117			
Parental obesity	Yes	51 (6.4)	751 (93.6)	291 (36.3)	294 (36.7)	166 (20.7)	51 (6.4)
	No	130 (4.5)	2743 (95.5)	1133 (39.4)	1042 (36.3)	568 (19.8)	130 (4.5)
	*P* value*	0.034		0.103			

PA: physical activity; ST: screen time; SES: socioeconomic status; MetS, Metabolic syndrome. * According to chi-squarer test

The adjusted and crude odds ratios (95% confidence intervals) for association between MetS and independent variables are presented in Table 3. Negative association was documented in the crude analysis between living in rural region and MetS (odds ratio [OR]: 0.57, 95% CI: 0.39-0.83), but this became non-significant after controlling for confounders (OR: 0.71, 95% CI: 0.46-1.12). According to crude OR, students with excess weight were 6.1 times more likely to have MetS (OR: 6.19, 95% CI: 3.55-10.79) and this association, although reduced, remained significant after adjustment for potential confounding factors (OR: ratio 4.24, 95% CI: 2.93-6.15). The association between healthy nutritional behavior and MetS was significant after adjusted potential confounders (OR: 0.62, 95% CI: 0.40-0.98). Students with high unhealthy nutritional behaviors showed increased risk of MetS in both crude (OR: 1.6, 95% CI: 1.05-2.44) and adjusted models (OR: 1.65, 95% CI: 1.05-2.63).

**Table 3:** Association of independent variables with metabolic syndrome in logistic regression model: the CASPIAN-V study

		**Crude**		**Adjusted**	
		**OR**	**95% CI**	** OR**	**95% CI**
Gender	Male	Reference	-	Reference	-
	Female	0.81	0.6-1.09	0.76	0.53-1.09
Age (year)	7-10	Reference	-	Reference	-
	11-14	1.07	0.76-1.52	1.04	0.68-1.58
	15-18	0.96	0.64-1.43	0.92	0.57-1.49
Region	Urban	Reference	-	Reference	-
	Rural	0.57	0.39-0.83*	0.71	0.46-1.12
PA	Low	Reference	-	Reference	-
	High	0.67	0.98-1.81	1.13	0.77-1.65
SES	Low	Reference	-	Reference	-
	Moderate	0.91	0.63-1.30	0.72	0.48-1.10
	High	0.85	0.58-1.2	0.68	0.44-1.04
ST	Low	Reference	-	Reference	-
	High	1.02	0.67-1.57	1.04	0.61-1.80
BMI	Normal weight	Reference	-	Reference	-
	Underweight	1.43	0.81-2.50	0.90	0.49-1.64
	Excess weight	6.19	3.55-10.79*	4.24	2.93-6.15*
Healthy nutritional behaviors	Low	Reference	-	Reference	-
	Moderate	0.69	0.46-1.04	0.62	0.40-0.98*
	High	0.82	0.56-1.20	0.83	0.55-1.26
Unhealthy nutritional behaviors	Low	Reference	-	Reference	-
	Moderate	1.47	0.96-2.25	1.49	0.94-2.36
	High	1.60	1.05-2.44*	1.65	1.05-2.63*
Parental obesity	No	Reference	-	Reference	-
	Yes	1.33	1.02-2.01	1.21	0.82-1.80

PA: physical activity; ST: screen time; SES: socioeconomic status; MetS, Metabolic syndrome; BMI, body mass index. * Statistically significant.

## Discussion

This nationwide study serves as confirmatory evidence on the importance of considering MetS in health issue of children and adolescents. The prevalence of MetS was high in urban, boys, and in those with obese parents.

Most of the previous studies in representative samples have demonstrated that MetS was more prevented in urban than rural areas.^11,12,27,28^ The lower prevalence of MetS in rural children might be because of their healthier lifestyle, in terms of higher physical activity and healthier dietary habits than their urban counterparts.

This study showed that parental obesity was associated with MetS of students. This finding was consistent with previous evidence that showed parental obesity is one of the risk factors.^29,30^ Children of obese parents are five times more likely to be obese.^31^ A study in Serbia reported higher maternal body weight in children and adolescents diagnosed with MetS.^32^ Family studies show that genetic factors account for about 50% of the variance in intra-abdominal fat even after controlling potential confounders for age, sex and total body fat.^33^ Other studies show that children of hypertensive parents also have more insulin resistance and higher blood pressure, serum cholesterol and triglyceride levels than controls.^34,35^ In Mexican Americans, parental type 2 diabetes is the most predictive factor for the development of MetS.^30^

We found that number of MetS components was correlated with male gender, higher age, urban residence and unhealthy dietary habits. Prevalence of MetS in adolescents was 13% including 21% of boys and 4% of girls in the United Arab Emirates.^36^

In the present study, children and adolescents with excess weight, i.e. obese and overweight, were 6.1 times more likely to have MetS which was consistent with previous studies.^37-41^

Our results found that consuming unhealthy nutritional behaviors was associated with increased risk of MetS.

A study of 14-year- old Australian adolescents found that Western dietary pattern was positively correlated with OR of MetS.^42^ A systematic review on 3168 Korean adolescents (13-18 years) between 1998-2009 showed that MetS was associated with western dietary pattern and traditional dietary pattern protectively associated with cardio metabolic risk factors.^43^ Positive association between energy-dense, high fat, low-fiber dietary patterns and fat mass index is documented,as well.^44^

In our study, sedentary life style was associated with MetS. This finding is line with previous evidence that screen time is associated with cardio-metabolic risk factors.^36,45^

The inverse association between PA and risk of MetS which was observed in current study was consistent with previous study which was performed in Danish pediatric population.^46^ In another study, lower PA was associated with MetS in both gender.^47^

One of the main limitation of this study is cross-sectional nature of study which preclude causal inference. Large and representative sample size are the main strengths of present study.

## Conclusion

The high prevalence of MetS and related risk factors in Iranian children and adolescents provides useful information for implementing effective preventive strategies based on diet, exercise, and life style modification rather than therapeutic modalities in later life.

## Ethical approval

This study was approved by ethical committee of Isfahan University of Medical Sciences.

## Competing interests

All authors declare no competing financial interests exist.

## Funding

Data of a national surveillance program, funded by the Ministry of Health, were used for the current study.

## Acknowledgments

The authors are thankful of all participants and large team working on this project in different provinces.
